# Dynamics of Aspergillus fumigatus in Azole Fungicide-Containing Plant Waste in the Netherlands (2016–2017)

**DOI:** 10.1128/AEM.02295-20

**Published:** 2021-01-04

**Authors:** Jianhua Zhang, Lidia Lopez Jimenez, Eveline Snelders, Alfons J. M. Debets, Anton G. Rietveld, Bas J. Zwaan, Paul E. Verweij, Sijmen E. Schoustra

**Affiliations:** a Laboratory of Genetics, Wageningen University, Wageningen, the Netherlands; b National Institute for Public Health and the Environment, Bilthoven, the Netherlands; c Department of Medical Microbiology, Radboud University Medical Centre, Nijmegen, the Netherlands; d Centre of Expertise in Mycology Radboudumc/CWZ, Radboud University Medical Centre, Nijmegen, the Netherlands; University of Illinois at Urbana-Champaign

**Keywords:** longitudinal study, azole resistance, triazoles, DMIs, *cyp51A*, plant waste stockpile, composting, long-term population dynamics, environmental heterogeneity

## Abstract

The treatment of patients suffering from *Aspergillus* diseases is hampered due to infections with Aspergillus fumigatus that are already resistant to medical azoles. Previous work has suggested that A. fumigatus likely gains resistance through environmental azole exposure in so-called hot spots. Here, we investigated A. fumigatus resistance dynamics over time at three sites at which farmers used azole fungicides for crop protection. Over 16 months, 114 samples were taken from stockpiles of decaying plant waste. A. fumigatus and azole fungicide residues were ubiquitously present in the plant waste. On average, 10^5^ A. fumigatus CFU/g was recovered, of which roughly half were itraconazole and tebuconazole resistant. Similar tandem repeat-mediated resistance mechanisms were found in colonies cultured from plant waste as reported in clinical azole-resistant isolates. Our results show a consistent high burden of azole-resistant A. fumigatus in azole-containing plant waste and underscores the need to further investigate resistance-reducing interventions and transmission routes.

**IMPORTANCE** Aspergillus fumigatus is consistently present independently on season at a high abundance in plant waste material throughout the sampling period. Our study confirmed that long-term storage sites of azole-containing decaying plant material can indeed be considered hot spots, which can sustain resistance development and maintenance in A. fumigatus. Roughly half of individual isolates were azole resistant and carried genetic mutations that are highly similar to those found in patients with azole-resistant invasive aspergillosis. Our work suggests that environmental sources of azole resistance in A. fumigatus may be important, underscoring the need for further studies on environment-to-patient transmission routes.

## Abstract (precis)

Aspergillus fumigatus is consistently present independently on season at a high abundance in plant waste material throughout the sampling period. Our study confirmed that long-term storage sites of azole-containing decaying plant material can indeed be considered hot spots, which can sustain resistance development and maintenance in A. fumigatus. Roughly half of individual isolates were azole resistant and carried genetic mutations that are highly similar to those found in patients with azole-resistant invasive aspergillosis. Our work suggests that environmental sources of azole resistance in A. fumigatus may be important, underscoring the need for further studies on environment-to-patient transmission routes.

## INTRODUCTION

Aspergillus fumigatus is a common plant waste-degrading fungus, of which spores are abundantly present in the air. When inhaled, these spores can cause diseases in animals and humans, ranging from allergic syndromes to acute invasive aspergillosis depending on the host immune system (1, 2). The number of drug classes that are available for the treatment of *Aspergillus* diseases remains very limited, with the azoles representing the major class. Azole resistance is an emerging concern in A. fumigatus, complicating the treatment of patients. Recent cohort studies indicate a 20% lower survival in patients with culture-positive voriconazole-resistant invasive aspergillosis than in patients with azole-susceptible infection (3, 4). Up to 90% of A. fumigatus isolates recovered from patients with azole-resistant invasive aspergillosis exhibit resistance mutations that are associated with resistance selection in the environment (5). These resistance mutations, e.g., TR_34_/L98H and TR_46_/Y121F/T289A, are believed to be selected through exposure to azole fungicides in the environment (6). Cross-resistance is commonly observed, and several azole fungicides have similar chemical structure as medical azoles (7). Furthermore, new resistance mutations continue to emerge in the environment (8), indicating that the diversity and frequency of resistance mutations in A. fumigatus are currently undersampled and are likely to continue to increase. Thus, exploring the potential and effectiveness of interventions in the environment is critical to reduce the resistance burden and safeguard the use of azoles for treating *Aspergillus* diseases. However, such interventions have to be informed by a better understanding of the dynamics of resistance selection in the environment (9, 10).

In previous research, we have gathered evidence that resistant A. fumigatus can accumulate and thrive in plant waste that contains environmental azoles (6, 8). The concept of a hot spot for azole-resistant A. fumigatus was postulated and is characterized by an environment that supports the growth and reproduction of A. fumigatus and where azole fungicides with anti-*Aspergillus* activity are present (6). In an initial survey, the following three hot spots were identified in the Netherlands: decaying plant waste of flower bulbs from farms where azoles are used, industrial wood-chopping waste, and industrial green-waste storage (6). The concept of a hot spot may be widely applicable and not limited to these three hot spots, and indeed, recently strawberry production waste was implicated as a hot spot for azole resistance selection in China (11). While taking into account the full cycle of fungicide application, plant waste collection, and composting, the stockpiling of plant waste that contained azole fungicides was found to be the phase associated with the highest burden of azole-resistant A. fumigatus (6). However, studies to date have used single time point sampling to investigate A. fumigatus resistance rather than studying its dynamics using repeated sampling over time. As stockpiles are kept for many months on farms, variations in weather conditions, i.e., temperature and humidity, might affect the burden of azole-resistant A. fumigatus within the stockpiles over the period that they are kept. Furthermore, the impact of other variables, such as the (chemical) persistence of azoles (their presence and activity), are poorly understood.

The present study aims to document and understand the dynamics of A. fumigatus populations within a hot spot in combination with the types and levels of fungicides that are present. Such insight is essential for designing effective measures. We performed a longitudinal study through repeated sampling of stockpiles of decaying plant waste from three farms where azoles were used for crop protection. Over a period of 16 months, a total of 114 samples were taken and analyzed. Specifically, we focused on trends over time regarding (i) the abundance of A. fumigatus, (ii) the fraction of A. fumigatus resistant to one representative environmental azole (tebuconazole [TEB]) and one representative medical azole (itraconazole [ITR]), (iii) the presence of azole fungicides and azole residues, and (iv) the genetic basis of resistance. As three independent sites were sampled over time, we further investigated variations between sampling sites.

## RESULTS

**FIG 1:**
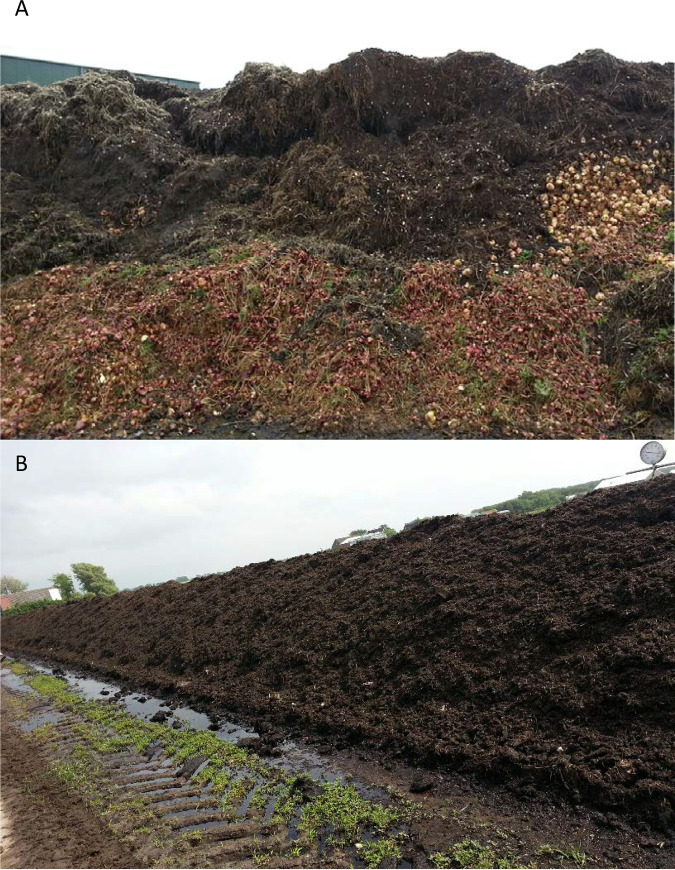
(A) An example of a stockpile of decaying plant waste where sampling took place. Heaps like this typically grow to 50 by 50 m wide and up to 10 m high. (B) The same material after various rounds of turning.

In total, 114 samples were taken from plant waste stockpiles (Fig. 1) between July 2016 and December 2017 at three flower bulb farms (A, B, and C). A detailed list of samples taken can be found in Table S1 in the supplemental material.

### Abundance of A. fumigatus throughout the sampling period.

**FIG 2:**
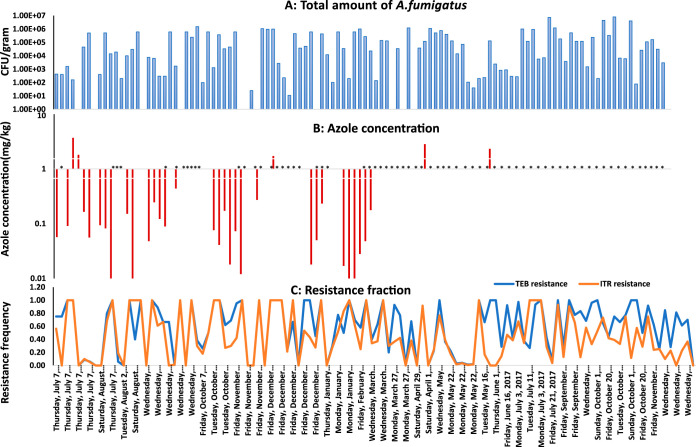
Overview of all samples taken over a period of 16 months. (A) Total abundance of A. fumigatus. (B) Azole concentration. *, samples that were not included in this analysis. (C) Resistance frequency to ITR and TEB.

**FIG 3:**
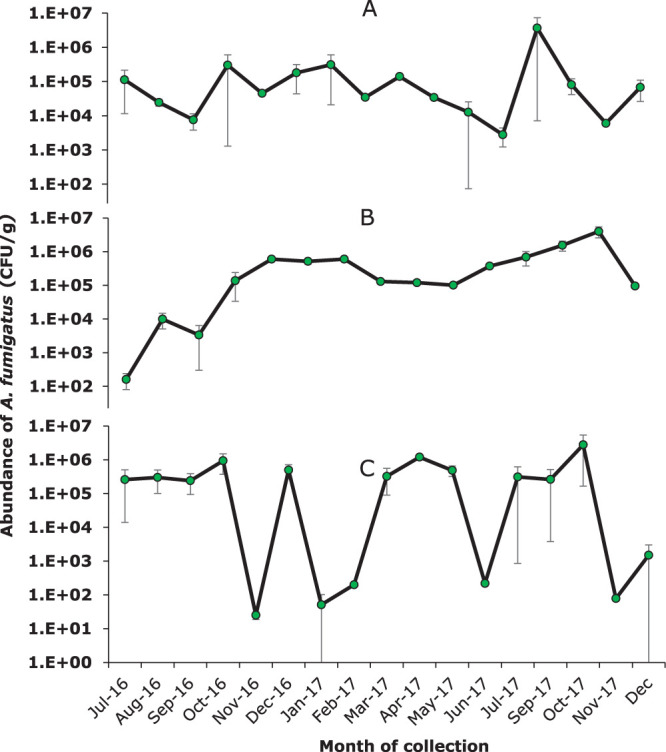
Abundance of A. fumigatus (in CFU/g) over a 16-month period at three sampling sites. Points show averages over all samples (vary from 2 to 5 replicates) of each sampling site collected in the same month. Error bars show standard error of the mean (SEM).

The total abundance of A. fumigatus in CFU per gram varied between the detection limit (10^1^) and 10^6^ CFU/g in the 114 samples, with an average of around 10^5^ CFU/g (Fig. 2 and 3). The density of A. fumigatus fluctuated throughout the sampling period and between sampling sites (Fig. 2 and 3). A total of 74% of the samples that were obtained contained high numbers (above 10^3^ CFU/g) of A. fumigatus CFU; 24% of the samples contained less than 10^3^ CFU/g. In 2% of the samples, A. fumigatus recovery was below the detection limit (which was 10^1^ CFU/g).

### Fraction of isolates resistant to indicator azole fungicides.

**FIG 4:**
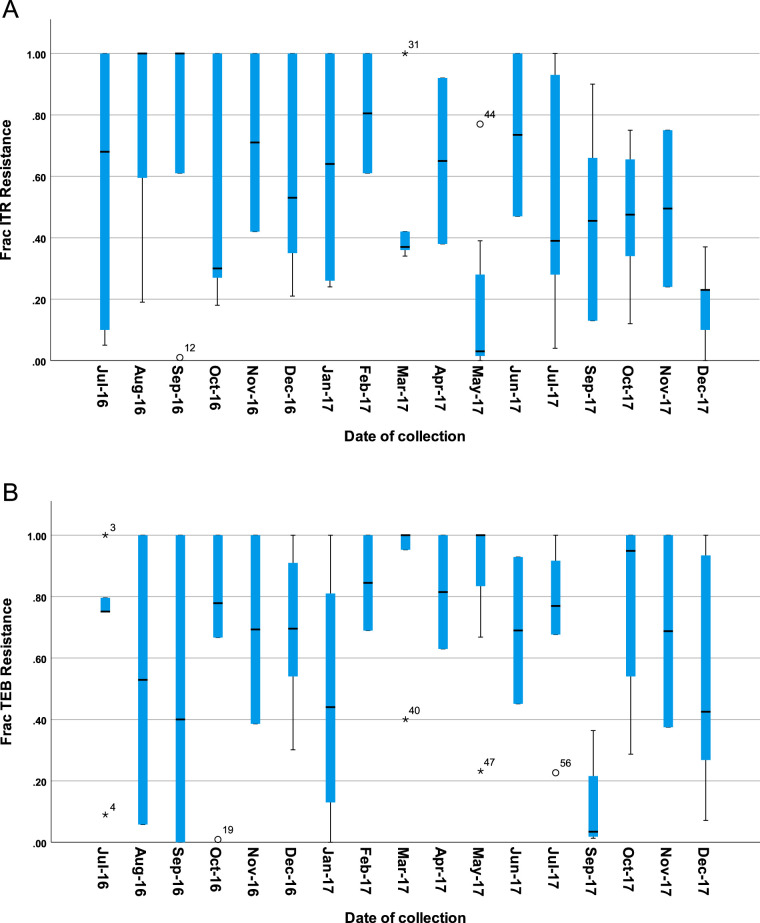
Fraction of resistant A. fumigatus against each of two indicator azole fungicides, the agricultural azole tebuconazole (TEB) and medical azole itraconazole (ITR) over a 16-month period. Points show that averages over all samples contained A. fumigatus assayed on each indicator azole for each sampling site taken in the same month. Error bars show confidence interval levels of 95%.

Both azole-resistant and azole-susceptible A. fumigatus colonies were recovered from all 114 samples. For both indicator azoles TEB and ITR, the fraction of resistant isolates fluctuated throughout the sampling period at around 50% (Fig. 2 and 4). Full results are shown in Table S1. There were no significant differences in fraction resistant over time or between sampling sites (chi-square test; *P* > 0.05).

The fraction of TEB-resistant isolates showed 90% overlap with ITR-resistant isolates, indicating that cross-resistance is the norm. However, in 10% of samples, cross-resistance between TEB and ITR was not observed. For example, in sample 89, the A. fumigatus population was ITR resistant while susceptible to TEB. In contrast, the A. fumigatus population in sample 21 was TEB resistant but ITR susceptible (Fig. 2; see also Table S1).

### Chemical analysis of samples taken.

For 37 representative samples selected from the entire sampling period across the three sampling sites, we determined the chemical profile including concentrations and types of azole fungicides. Fungicides were detected in all samples, ranging between 3 and 14 different types of fungicide (see Table S2 in the supplemental material). In our analysis, we focused on the triazoles, which show activity against A. fumigatus (7). In all samples, prothioconazole-desthio, TEB, and thiabendazole were detected at concentrations ranging from 0.01 to 3.7 mg/kg. The azole fungicide concentrations fluctuated over the sampling period (Fig. 2; see also Table S2). The majority (86%, 32/37) of samples contained azole fungicide concentrations of <1 mg/kg, while 14% (5/37) of samples contained >1 mg/kg (Fig. 2; see also Table S2).

### Genetic basis of resistance: changes in the *cyp51A* gene and its promoter region.

We selected 117 TEB- and/or ITR-resistant A. fumigatus isolates that were representative for the different time points and sampling sites for genetic analysis. The isolates were taken from samples with abundant A. fumigatus growth (>10^2^ CFU/g). Table 1 shows the summarized results, and full details can be found in Table S3 in the supplemental material. Mutations in the promoter region and coding region of the *cyp51A* gene were found in all 117 azole-resistant isolates. Seven different genetic types were detected among the resistant strains, including the commonly found resistance genotypes TR_34_/L98H and TR_46_/Y121F/T289A. For strains containing TR_46_, point mutations in the coding part of the gene (I364V and S363P) were observed, of which the significance for the azole-resistant phenotype has not been documented.

**TABLE 1:** Genetic analysis of the *cyp51A* promoter region and structural gene of 117 representative azole-resistant A. fumigatus isolates per sampling site

Tandem repeat (promoter region)	Point mutation(s) (coding gene)	No. per sampling site			Total no.
		A	B	C	
TR_34_	L98H	7	2	8	17
TR_34_	L98H/S297T	1	1	0	2
TR_46_	Y121F/T289A	24	23	33	80
TR_46_	Y121F/M172I/T289A/G448S	2	0	2	4
TR_46_	Y121F/T289A/S363P/I364V/G448S	1	8	0	9
TR_46_^3^	Y121F/M172I/T289A/G448S	0	2	2	4
TR_46_^4^	Y121F/M172I/T289A/G448S	0	1	0	1
Total no.					117

**FIG 5:**
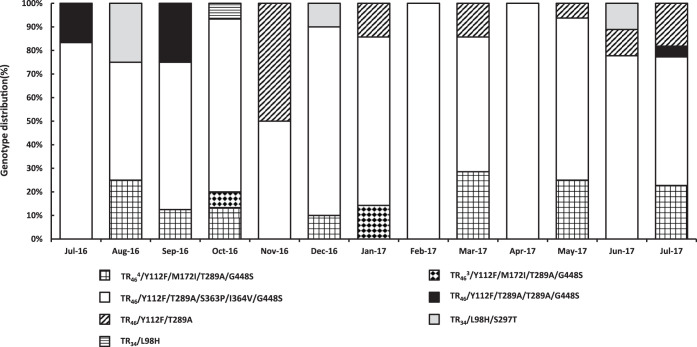
The distribution of different *cyp51A* gene resistance genotypes in A. fumigatus over a 1-year period for locations A, B, and C.

For the 117 resistant isolates, the TR_46_-derived mutations accounted for 79% followed by TR_34_-derived mutations (16%). TR_46_^3^- and TR_46_^4^-derived mutations were present the least common (∼5%) (Table 1). At sampling site B, a larger variety of resistance mechanisms in the samples were found (TR_34_, TR_46_, TR_46_^3^, and TR_46_^4^) compared with the other two sites, where only TR_34_ and TR_46_ mutations were recovered (Table 1). TR_46_-derived mutations (TR_46_/Y121F/T289A) were dominant over the whole sampling period. There were no trends observed in the diversity of resistance mechanisms over time (Fig. 5).

**FIG 6:**
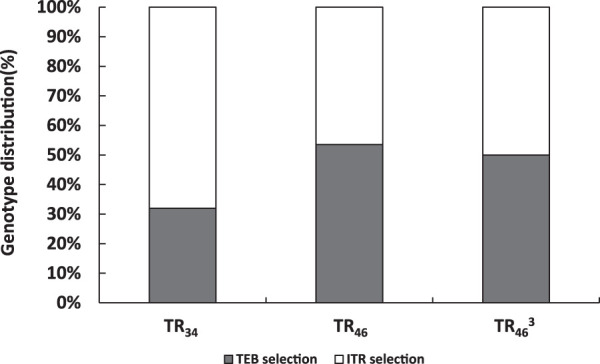
Distribution of resistant A. fumigatus isolates retrieved from medical itraconazole (ITR) selection and agricultural tebuconazole (TEB) selection medium for locations A, B, and C combined.

No notable differences were found in distribution of resistance mutations (TR_46_ and TR_46_^3^) according to the azole selection medium used for isolation of the strains (Fig. 6). For TR_46_/Y121F/T289A, 45% of isolates were cultured from ITR selection plates and 54% from TEB plates. For isolates harboring TR_46_^3^-derived mutations, 50% were cultured from ITR plates and 50% from TEB selection plates. While for TR_34_/L98H, 32% of isolates were recovered from ITR-plates and 68% from TEB plates.

We selected a representative sample of 32 isolates, including all resistance mechanisms, by choosing up to 3 isolates per farm per resistance genotype for *in vitro* susceptibility testing using the EUCAST broth microdilution reference assay. The azole phenotypes are consistent with previously reported characteristics, i.e., highly ITR resistant in TR_34_ variants and highly voriconazole resistant in TR_46_ variants (Table 2). However, variations in activity of other azoles was observed in isolates harboring the same resistance mutation.

**TABLE 2:** *In vitro* activity of ITR, voriconazole, and posaconazole against a selection of 32 A. fumigatus isolates

Strain	Sampling site	*cyp51A* gene genotype	MIC (mg/liter)^*a*^		
			ITR	VOR	POSA
ATCC 204305		Wild type	0.125	0.25	0.03
11-1	A	TR_34_/L98H	>16	2	0.5
37-1	A	TR_34_/L98H	>16	8	1
66-4	A	TR_34_/L98H	>16	8	1
19-3	B	TR_34_/L98H	>16	16	1
26-4	B	TR_34_/L98H	>16	>16	0.5
55-3	C	TR_34_/L98H	>16	2	0.5
78-2	C	TR_34_/L98H	>16	4	0.5
84-2	C	TR_34_/L98H	>16	4	1
47-4	A	TR_34_/L98H/S297T	>16	16	0.5
39-3	B	TR_34_/L98H/S297T	>16	1	0.5
5-1	A	TR_46_/Y121F/T289A	0.5	>16	0.25
11-2	A	TR_46_/Y121F/T289A	16	>16	0.5
18-4	A	TR_46_/Y121F/T289A	>16	>16	0.5
13-3	B	TR_46_/Y121F/T289A	2	>16	1
13-4	B	TR_46_/Y121F/T289A	1	>16	1
19-2	B	TR_46_/Y121F/T289A	1	>16	0.5
26-1	B	TR_46_/Y121F/T289A	0.5	>16	0.25
8-3	C	TR_46_/Y121F/T289A	2	>16	0.5
25-3	C	TR_46_/Y121F/T289A	>16	>16	0.5
38-3	C	TR_46_/Y121F/T289A	2	>16	0.5
18-1	A	TR_46_/Y121F/M172I/T289A/G448S	2	>16	0.5
18-3	A	TR_46_/Y121F/M172I/T289A/G448S	1	>16	0.5
13-2	B	TR_46_^3^/Y121F/M172I/T289A/G448S	1	>16	0.5
8-2	C	TR_46_/Y121F/M172I/T289A/G448S	1	>16	0.25
102-2	C	TR_46_/Y121F/M172I/T289A/G448S	1	>16	0.5
40-1	A	TR_46_/Y121F/T289A/S363P/I364V/G448S	2	>16	1
52-2	B	TR_46_/Y121F/T289A/S363P/I364V/G448S	>16	>16	1
70-1	B	TR_46_/Y121F/T289A/S363P/I364V/G448S	2	>16	1
77-3	B	TR_46_/Y121F/T289A/S363P/I364V/G448S	1	>16	1
62-4	C	TR_46_^3^/Y121F/M172I/T289A/G448S	>16	>16	1
92-1	C	TR_46_^3^/Y121F/M172I/T289A/G448S	2	>16	1
39-2	B	TR_46_^4^/Y121F/M172I/T289A/G448S	2	>16	0.5

a VOR, voriconazole; POSA, posaconazole.

### Correlations among the different environmental parameters.

Due to the fact that data does not follow a normal distribution, we performed a series nonparametric Spearman correlation tests, testing for correlations among the different environmental parameters. We found mostly weak and nonsignificant pairwise correlations between azole levels, total CFU, resistance frequency (to TEB and ITR), sample collection date, and farm site. Exception was the strong correlation between TEB resistance frequency (TEBf) and ITR resistance frequency (ITRf) (correlation coefficient = 0.904; *P* = 0.023) (see Table S4 in the supplemental material).

## DISCUSSION

This study describes the results of longitudinal sampling of stockpiles of azole-containing plant waste, which was previously identified as a hot spot for resistance selection (6). In our analysis, we focused on the abundance of A. fumigatus, the fraction of A. fumigatus resistant to azole fungicides, the presence of azole fungicides and azole residues, and the genetic basis of resistance. Our results show that the sites we sampled can indeed be considered hot spots. The long-term storage of azole-containing decaying plant material can sustain resistance development and maintenance, supporting previous findings (8).

Specifically, our results show that A. fumigatus was consistently present throughout the sampling period at the three sampling sites at levels around 10^5^ CFU/g. CFU levels appear to be little affected by the local climate or weather conditions. The average temperature of the Netherlands is about 2°C (35°F) in January and 19°C (66°F) in July, with an annual average temperature of approximately 10°C (50°F). Moreover, similar A. fumigatus densities were present at the different sampling sites. In estimation, a single stockpile with dimensions 50 by 50 by 10 m may contain as many as 2.5 × 10^15^ spores, which underscores the substantial burden that the A. fumigatus spores may cause. The A. fumigatus concentrations that we observed were higher than those reported in a study involving environmental substrates collected in Italy, Spain, Hungary, and Germany (12). In these samples, the highest A. fumigatus concentrations were found in corn silage samples after 14 days of air exposure, ranging between 7.4 × 10^4^ and 23.5 × 10^6^ CFU/g (12). In other compost types involving different starting material and undergoing varying processing histories, lower densities were found ranging from 1.0 × 10^2^ up to 1.1 × 10^4^ CFU/g (12, 13). In conventional agricultural fields in Germany with fungicide exposure, only 10^0^ to 10^2^ CFU/g A. fumigatus was found. In our azole-containing compost, we observed in total an average of 10^4^ CFU/g resistant A. fumigatus throughout the 16-month sampling period in azole-containing decaying plant material. This is in contrast to studies where samples were taken at a location without azole pressure, where 10^3^ CFU/g of resistant A. fumigatus was found (8). In German organic fields without fungicides exposure, 0 to 1 CFU/g displayed resistance to medical azoles (14). Thus, in our study, we provide data backing up previous suggestions that azole-containing decaying plant material is a reservoir and selective environment for resistant A. fumigatus.

Among the A. fumigatus isolates recovered from the stockpiles, roughly half were resistant to our two indicator azole fungicides TEB and ITR. The fraction of resistance to the two azoles used for testing was highly similar, suggesting that agricultural azoles can induce or at least support cross-resistance to medical triazoles, confirming laboratory studies (15). The presence of both susceptible and resistant A. fumigatus may indicate variations in azole selection pressure in microenvironments within the stockpiles. Indeed, measurements of concentrations of fungicides present in the waste material indicated notable variation both regarding fungicide compound and concentration. Factors such as addition of fresh bulb waste material or wash out of azoles during rainfall will greatly impact these microenvironmental conditions. The absence of significant correlations between environmental parameters, such as azole concentration, total CFU, and resistance mechanism, could be explained by the heterogeneity of the compost.

The discovery of large genetic variation regarding the resistance mechanism in our samples suggests that azole-containing plant waste can support a wide range of various resistant strains. Notable variation was also observed for the azole phenotypes. Even isolates harboring identical resistance mutations showed variable resistance phenotypes, which might indicate that isolates with tandem repeat (TR)-mediated resistance may harbor additional resistance mechanisms. Thus, plant waste material is highly heterogeneous and supports a widely diverse population of A. fumigatus; this heterogeneity and/or other factors allow the different genotypes to coexist in the same heap and new genotypes to appear. How these new genotypes are generated and what the genetic relationship between these different genotypes is needs further research.

Genetic analysis of resistant A. fumigatus isolates showed a striking similarity with those commonly reported in clinical A. fumigatus isolates. A recent analysis of the Dutch national resistance surveillance program, which included 640 azole-resistant A. fumigatus isolates obtained from 508 patients, showed TR_34_ and TR_46_ mutations in 86% of resistant isolates (5). Interestingly, some of the clinical isolates harbored polymorphisms in the *cyp51A* gene that are similar to those found in the stockpiles, for instance, S297T in isolates harboring TR_34_/L98H. This observation supports a link between environmental resistance and human infection. However, it remains unclear how azole-resistant A. fumigatus is transmitted from an environmental source to the patient and which proportion of clinical isolates can be traced to flower bulb hot spots. There are several observations that underscore the need to investigate transmission routes of resistant A. fumigatus. First, the distribution of TR_34_ and TR_46_ mutations over the samples shows an opposite pattern in the stockpile and clinical isolates. While we found 83.7% TR_46_ mutations in isolates obtained from the stockpiles, these mutations were concurrently found in only 17% of azole-resistant clinical A. fumigatus isolates (5). This might suggest that other sources of azole-resistant A. fumigatus play a greater role in transmission to humans or that there is certain selection for entrance to the lung. Conversely, the distribution of A. fumigatus isolates in stockpiles and patients may also be asynchronously correlated. Furthermore, several studies have investigated the risk of bioaerosol exposure from composting facilities on human health, especially respiratory illnesses (16). A. fumigatus is a well-described and common component of bioaerosols and, thus, is considered a good indicator for bioaerosol emissions in epidemiological studies (17). Although high A. fumigatus aerial dispersal rates are found when turning compost heaps (18), epidemiologic studies find no evidence for a relation between bioaerosol exposure and hospital admissions for respiratory disease (17). To further understand the significance of azole-resistant A. fumigatus in (flower bulb) compost and human infection, high-resolution genotyping of environmental and clinical A. fumigatus isolates is needed. Such a study is under way, taking into account the geographic proximity to potential hot spots. In addition, the search for other hot spots in the environment needs to be continued.

In summary, our findings support the notion that heaps of decaying plant material that contain azoles are important hot spots for the development and maintenance of azole-resistant A. fumigatus in the environment. Future work should investigate what variations in plant waste materials and its storage affect A. fumigatus abundance and resistance. A laboratory study mimicking the field conditions can help elucidate mechanisms that support (resistant) A. fumigatus under these conditions. Further, especially various ways of processing plant waste from stockpiles to mature compost (19) should be systematically studied since these processing techniques may greatly reduce the abundance of A. fumigatus. Finally, while the similarity of genetic mechanisms of resistance exist between the environmental isolates from our study and those isolated from patients, the significance of this finding for human infection remains unclear, as transmission routes are largely unexplored. Future work should also focus on transmission routes, as these insights are critical to design effective interventions.

## MATERIALS AND METHODS

### Sampling sites.

**FIG 7:**
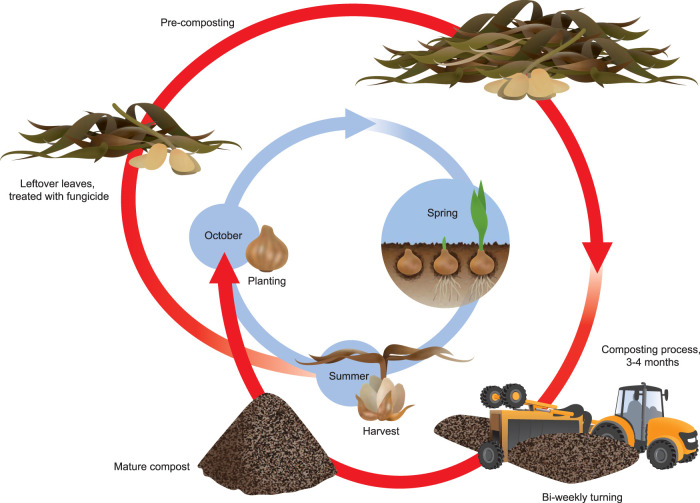
The process from collection of plant waste to mature compost. During the cultivation of the crops, azoles are used for crop protection. From July on, after harvesting the bulbs, inappropriately sized bulbs and leftover leaves are collected as waste, which is added to a stockpile serving as storage heap. By April of the next year, the heap is around 6 to 10 m high and around 50 by 50 m wide. Then, formal aerobic composting starts with mixing the accumulated plant-decaying materials with straw to insert oxygen and regularly turning the mixture for six or seven rounds until June, after which composting is completed.

Sampling was performed at three flower bulb farms (labeled A, B, and C in the same geographical region) that used azole fungicides for crop protection (against, for instance, *Fusarium* and *Botrytis*) and that stored plant waste on site in stockpiles. All three farms use common conventional methods for crop protection, which includes antifungal azole application. From July, plant waste is collected from the fields, and a stockpile is built to which material is added over the course of the year. After several months, the stockpile heap can be 6 to 10 m high and around 50 by 50 m wide. Although some decomposition of organic material takes place during stockpiling, its efficiency is limited by lack of oxygen. Therefore, to increase the decomposition rate, active management of the stockpile through turning is required to maintain sufficient supply of oxygen and adequate moisture levels. Commonly, in April of the next year, active composting is initiated by the farmer by inserting straw and regular turning of the pile in six to seven rounds. In June, the multistep composting process is completed (Fig. 7).

### Sampling.

Sampling was performed between July 2016 and December 2017. We sampled from growing stockpiles of plant waste before processing to mature compost since we previously found evidence that A. fumigatus CFU in mature compost were reduced to below detectable limits and no azole-resistant A. fumigatus in mature compost was detected (6). Between two and five samples were taken at each of the three sampling sites every month at various places in the growing stockpile heap (see Table S1 in the supplemental material). Per sample, approximately 10 g of material was collected and stored at 4°C for further analysis.

### A. fumigatus culturing and resistance testing.

For each sample of organic material, 5 g was added to 10 ml sterile saline (0.8 g/liter NaCl in water) with 0.05% Tween and diluted. Then, 50 μl of the diluted suspension (10^0^ to approximately 10^3^ until there are countable colonies on the plates) was plated on malt extract agar (MEA) supplemented with streptomycin (10 μg/ml) and tetracycline (15 μg/ml) (Sigma-Aldrich, Germany) to suppress growth of bacteria. Cultures were incubated at 48°C, which is selective for A. fumigatus growth (20). After 2 days of incubation, colonies were counted to estimate A. fumigatus density. Colonies that showed atypical *Aspergillus* morphology were selected and verified by amplifying (PCR) and sequencing part of the β-tubulin and carboxypeptidase-5 genes (8, 20). Further, we plated the same diluted suspension, which gave countable colonies on MEA plates supplemented with one of the following two indicator azoles: the azole fungicide TEB (tebuconazole) (Sigma-Aldrich, Germany) or the medical azole ITR (itraconazole) (Sigma-Aldrich, Germany), both at 4 mg/liter (6). Resistance was defined as the ability to grow on either of these agar plates. After 4 days of incubation at 48°C, the number of resistant colonies was documented, and the percentage of resistant strains of the total number of A. fumigatus strains that had been isolated was calculated. Two resistant phenotypically different A. fumigatus colonies from each plate were stored in glycerol at −80°C for further analyses.

*In vitro* susceptibility testing was performed on a subset of isolates using the EUCAST broth microdilution reference method and the medical azoles ITR, voriconazole, and posaconazole (21). Reference control strain ATCC 204305 was included in the assay.

### Screening for *cyp51A* mutations in azole-resistant colonies.

We characterized TEB- and/or ITR-resistant isolates for their *cyp51A* gene, focusing on tandem repeat (TR) variation in the promoter region and point mutations in the coding gene commonly found to confer resistance. From representative samples, up to three single resistant colonies recovered from −80°C were subjected to a DNA extraction protocol (19). The full coding sequences of the *cyp51A* gene and the promoter region were screened by PCR amplification and subsequent sequencing as previously described (22, 23). The *cyp51A* sequence (GenBank accession number AF338659) was used as a reference to detect mutations.

### Analysis of fungicide concentrations in samples of decaying plant waste.

In order to assess which fungicides were present in the samples and how the fungicide concentrations fluctuate over time, we selected 37 representative samples over the 16-month sampling period for chemical analysis of azole fungicides and azole residues (24). The methods used were gas chromatography-tandem mass spectrometry (GC-MS/MS) and liquid chromatography-tandem mass spectrometry (LC-MS/MS) by the Eurofins laboratory (https://www.eurofinsfoodtesting.nl/).

### Statistical analyses.

Data distribution analyses were performed via SPSS Analyze, Descriptive statistics, Explore plots, Histogram. Correlation tests among total CFU, resistance frequency to TEB and ITR, resistance type, and azole concentration in the compost samples were performed via SPSS Correlate, Bivariate, Spearman.

### Data availability.

Sequence data can be found at https://datadryad.org/stash/share/0O43SjgdCVeh_6Zt2cND05hLDVRajYaFsDzqIICwzdQ. All isolated strains will be made available upon request.

## Supplementary Material

Supplemental file 1
