# Supplementary material for: Dynamics of Aspergillus fumigatus in Azole Fungicide-Containing Plant Waste in the Netherlands (2016–2017)
Source: Appl Environ Microbiol. 2021 Jan 4;87(2):e02295-20. doi: 10.1128/AEM.02295-20 (PMC7783342; doi:10.1128/AEM.02295-20)
Supplement: Supplemental file 1 [file AEM.02295-20-s0001.pdf]

Supplementary Table 1. Overview of samples taken and their analysis.

Here we report the sampling date, location, abundance of *A. fumigatus*, fraction resistant isolates to TEB and ITR and the concentration of azoles. For samples where the abundance of *A. fumigatus* was below the detection limit of 10^1 (the detection level), the abundance was scored as 0 (zero). ND: not determined.

Sample number	Date of collection	Sampling sites	Total *A. fumigatus* (CFU/g)	Resistance fraction		Triazoles concentration(mg/kg)
				TEB	ITR	
1	7-Jul-16	A	4.26E+02	0.75	0.56	0.057
2	7-Jul-16	A	4.00E+02	0.75	0.00	ND
3	7-Jul-16	A	1.60E+03	1.00	1.00	0.091
6	7-Jul-16	B	1.60E+02	1.00	1.00	3.708
7	7-Jul-16	B	0.00E+00	0.00	0.00	1.816
4	7-Jul-16	A	4.47E+04	0.09	0.10	0.165
5	7-Jul-16	A	5.15E+05	0.80	0.68	0.082
8	7-Jul-16	C	5.04E+05	0.06	0.05	0.056
9	7-Jul-16	C	1.40E+04	1.00	1.00	0.01
10	2-Aug-16	C	0.00E+00	0.00	0.00	ND
11	2-Aug-16	A	1.90E+04	0.06	0.19	ND
12	2-Aug-16	B	2.00E+02	0.00	0.00	ND
13	20-Aug-16	B	9.90E+03	1.00	1.00	0.152
14	20-Aug-16	A	3.00E+04	0.40	1.00	0.01
15	20-Aug-16	C	6.00E+05	1.00	1.00	0
16	20-Aug-16	C	4.00E+02	0.00	0.00	0.094
17	14-Sep-16	A	0.00E+00	0.00	0.00	ND
18	14-Sep-16	A	7.60E+03	1.00	1.00	0.048
19	14-Sep-16	B	6.40E+03	0.89	0.61	0.246
20	14-Sep-16	B	3.00E+02	0.67	0.67	0.122
21	14-Sep-16	C	3.00E+02	0.67	0.00	0.089
22	14-Sep-16	C	6.00E+05	0.01	0.01	ND
23	14-Sep-16	C	1.70E+03	1.00	1.00	0.44
24	14-Sep-16	C	0.00E+00	0.00	0.00	ND
25	14-Sep-16	C	6.00E+05	1.00	1.00	ND
26	6-Oct-16	B	2.41E+05	0.39	0.30	ND
27	7-Oct-16	C	1.50E+06	0.26	0.18	ND
31	11-Oct-16	C	1.00E+02	0.50	0.50	ND
32	11-Oct-16	A	6.00E+05	1.00	1.00	ND
37	25-Oct-16	A	1.30E+03	1.00	1.00	0.076
38	25-Oct-16	C	3.71E+05	0.62	0.27	0.041
39	16-Oct-16	B	3.34E+04	0.69	0.30	0.172
40	18-Nov-16	A	4.51E+04	0.95	0.42	0.018
41	18-Nov-16	B	6.00E+05	1.00	1.00	0.073
42	18-Nov-16	C	0.00E+00	0.00	0.00	0.012
43	18-Nov-16	C	0.00E+00	0.00	0.00	ND

44	18-Nov-16	C	2.50E+01	1.00	1.00	ND
45	18-Nov-16	C	0.00E+00	0.00	0.00	0.272
60	9-Dec-16	C	1.05E+06	1.00	1.00	ND
61	9-Dec-16	C	9.50E+05	1.00	1.00	ND
62	9-Dec-16	C	9.80E+05	1.00	1.00	1.721
63	9-Dec-16	C	2.70E+03	0.23	0.21	ND
64	9-Dec-16	C	2.26E+02	0.67	1.00	ND
65	9-Dec-16	C	1.10E+01	0.00	0.00	ND
66	9-Dec-16	A	4.50E+05	1.00	0.53	ND
67	9-Dec-16	A	3.70E+04	1.00	0.43	ND
68	9-Dec-16	A	5.10E+04	0.45	0.27	ND
46	5-Jan-17	A	6.00E+05	1.00	1.00	0.018
49	5-Jan-17	C	0.00E+00	0.00	0.00	0.05
52	5-Jan-17	B	4.32E+05	0.30	0.28	0.233
47	30-Jan-17	A	1.20E+04	0.78	0.52	ND
50	30-Jan-17	C	1.02E+02	0.50	0.83	ND
53	30-Jan-17	B	6.00E+05	1.00	1.00	ND
48	24-Feb-17	A	3.45E+04	0.70	0.61	0.017
51	24-Feb-17	C	2.00E+02	0.58	0.25	0.01
54	24-Feb-17	B	6.00E+05	1.00	1.00	0.01
55	8-Mar-17	C	1.00E+06	0.40	0.34	0.028
56	8-Mar-17	C	2.70E+05	0.63	0.37	0.048
57	8-Mar-17	C	2.30E+04	1.00	1.00	0.177
58	8-Mar-17	C	1.40E+02	0.20	0.30	ND
69	27-Mar-17	A	1.40E+05	0.93	0.36	ND
70	27-Mar-17	B	1.30E+05	0.77	0.42	ND
71	27-Mar-17	C	0.00E+00	0.00	0.00	ND
72	29-Apr-17	A	3.40E+04	0.68	0.38	ND
73	29-Apr-17	B	0.00E+00	0.00	0.00	ND
74	29-Apr-17	C	1.20E+06	0.92	0.92	ND
75	1-Apr-17	C	0.00E+00	0.00	0.00	ND
89	1-May-17	A	4.00E+01	1.00	0.00	ND
76	10-May-17	A	3.80E+04	0.23	0.24	ND
77	10-May-17	B	1.20E+05	1.00	0.77	2.836
78	10-May-17	C	1.10E+06	0.36	0.39	ND
90	16-May-17	A	1.07E+02	0.66	0.17	ND
81	22-May-17	C	5.10E+05	0.22	0.17	ND
82	22-May-17	C	7.60E+05	0.03	0.02	ND
83	22-May-17	C	4.00E+05	0.04	0.04	ND
87	22-May-17	C	1.30E+05	0.01	0.01	ND
88	22-May-17	C	1.40E+04	0.02	0.02	ND
91	1-Jun-17	B	7.20E+04	1.00	1.00	ND
92	1-Jun-17	C	2.00E+02	1.00	0.00	ND
93	16-Jun-17	C	2.37E+02	0.29	0.14	ND
94	16-Jun-17	B	1.30E+05	0.92	0.47	2.33

95	3-Jul-17	A	2.40E+03	0.42	0.39	ND
96	3-Jul-17	C	8.25E+02	0.97	0.68	ND
98	11-Jul-17	C	8.89E+02	0.37	0.34	ND
100	11-Jul-17	A	2.91E+02	0.27	1.00	ND
101	21-Jul-17	B	2.73E+02	0.93	1.00	ND
97	3-Jul-17	B	1.00E+06	1.00	1.00	ND
99	11-Jul-17	B	1.20E+05	0.43	0.28	ND
102	21-Jul-17	C	9.30E+05	0.07	0.04	ND
103	21-Jul-17	A	5.70E+03	1.00	0.93	ND
104	15-Sep-17	A	7.10E+03	0.30	0.13	ND
105	6-Sep-17	A	7.30E+06	1.00	0.90	ND
106	15-Sep-17	B	1.20E+06	0.78	0.66	ND
107	6-Sep-17	B	1.80E+05	0.83	0.13	ND
108	6-Sep-17	C	3.80E+03	0.68	0.58	ND
109	15-Sep-17	C	5.20E+05	0.96	0.33	ND
110	1-Oct-17	A	1.20E+05	1.00	0.53	ND
111	10-Oct-17	A	1.20E+05	0.65	0.73	ND
112	20-Oct-17	A	1.50E+03	0.44	0.42	ND
113	1-Oct-17	B	2.40E+05	0.75	0.39	ND
114	10-Oct-17	B	2.00E+02	0.67	0.33	ND
115	20-Oct-17	B	4.40E+06	0.77	0.75	ND
116	1-Oct-17	C	3.30E+05	1.00	0.12	ND
117	10-Oct-17	C	8.00E+06	1.00	0.58	ND
118	20-Oct-17	C	6.60E+03	0.50	0.29	ND
119	10-Nov-17	A	6.00E+03	0.92	0.75	ND
120	10-Nov-17	B	4.00E+06	0.63	0.24	ND
121	10-Nov-17	C	7.80E+01	0.25	0.25	ND
122	6-Dec-17	A	2.60E+04	0.85	0.10	ND
123	13-Dec-17	A	1.10E+05	0.28	0.23	ND
124	6-Dec-17	B	1.60E+05	0.81	0.00	ND
125	13-Dec-17	B	3.10E+04	0.61	0.23	ND
126	6-Dec-17	C	3.00E+03	0.70	0.37	ND
127	13-Dec-17	C	0.00E+00	0.00	0.00	ND

Supplementary Table 2. Chemical analysis of samples

Here we report the concentrations of azoles and their residues as well as concentrations of other compounds that were detected. Azoles are indicated in bold and red.

**Sample number**	**Fungicides type**	**(mg/kg)**	**Azole fungicides amount(mg/kg)**
Sample 1	**Tebuconazole**	0.016	**0.057**
	**Prothioconazole-destio**	0.041	
	Boscalid	0.017	
	Fluopyram	0.052	
	Flutolanil	0.024	
	Prochloraz	0.10	
Sample 3	**Prothioconazole-desthio**	**0.078**	**0.091**
	**Tebuconazole**	**0.013**	
	Pendimethalin	0.046	
	Chloorprofam	0.048	
	Quintozeen	0.021	
	Bixafen	0.01	
	Boscalid	0.014	
	Butocarboxim sulfoxide	0.11	
	Carbendazim	0.011	
	Dimethoaat	0.1	
	Fluopyram	0.024	
	Flutolanil	0.023	
	Prochloraz	0.087	
	Anthrachinon	0.028	
Sample 4	**Prothioconazole-desthio**	**0.130**	**0.165**
	**Tebuconazole**	**0.035**	
	Captan (som)	0.76	
	Chloorthalonil	0.065	
	Folpet (som)	1.2	
	Pendimethalin	0.06	
	Pirimifos-methyl	0.013	
	Procymidon	0.092	
	Carbendazim (som)	0.38	
	Fluazinam	0.32	
	Prochloraz (som)	1.3	
	Pyraclostrobine	1.5	
	Thiofanaat-methyl	0.52	
Sample 5	**Prothioconazole-desthio**	0.022	**0.082**
	**Tebuconazole**	0.06	
	Prochloraz (som)	2.9	

	Captan (som)	0.31	
	Chloorthalonil	0.4	
	Folpet (som)	0.19	
	Procymidon	0.014	
	Carbendazim (som)	1.1	
	Fluazinam	2.6	
	Imidacloprid	4.9	
	Pyraclostrobine	2.9	
Sample 6	**Prothioconazole-desthio**	3.2	**3.708**
	**Tebuconazole**	0.42	
	**Thiabendazole**	0.088	
	Prochloraz (som)	8.3	
	Captan (som)	8.6	
	Chloorprofam (som)	0.047	
	Chloorthalonil	0.98	
	Dicofol	0.011	
	Fludioxonil	0.043	
	Folpet (som)	3.1	
	Metolachloor-S	0.18	
	Pendimethalin	1.0	
	Pirimifos-methyl	0.071	
	Procymidon	0.046	
	Abamectine (som)	0.011	
	Boscalid	0.042	
	Carbendazim (som)	1.1	
	Cyprodinil	0.014	
	Fluazinam	3.8	
	Flutolanil	0.011	
	Metamitron	0.14	
	Pyraclostrobine	5.6	
	Thiofanaat-methyl	17.0	
	Tolclofos-methyl	0.013	
Sample 7	**Prothioconazole-desthio**	1.8	**1.816**
	**Tebuconazole**	0.016	
	Prochloraz	0.98	
	Captan (som)	73.0	
	Chloorthalonil	0.016	
	Metolachloor-S	0.034	
	Pendimethalin	0.046	
	Pirimifos-methyl	0.073	
	Procymidon	0.015	
	Carbendazim	0.35	

	Cyprodinil	0.016	
	Fluazinam	19.0	
	Imidacloprid	0.047	
	Pyraclostrobine	0.35	
	Thiofanaat-methyl	22.0	
Sample 8	**Prothioconazole-desthio**	0.04	**0.056**
	**Tebuconazole**	0.016	
	Prochloraz	2.9	
	Captan	0.14	
	Chloorprofam	0.013	
	Folpet	0.032	
	Boscalid	0.032	
	Carbendazim	0.83	
	Fluazinam	0.24	
	Fluopyram	0.011	
	Pyraclostrobine	0.083	
	Spirotetramat	0.044	
Sample 9	**Prothioconazole-desthio**	0.01	0.010
	Prochloraz	1.6	
	Captan	0.16	
	Chloorthalonil	21.0	
	Folpet	0.62	
	Quintozeen	0.024	
	Carbendazim	0.73	
	Fluazinam	0.52	
	Fluopyram	0.022	
	Imidacloprid	0.93	
	Pyraclostrobine	3.2	
	Thiofanaat-methyl	0.12	
Sample 13	**Prothioconazole-desthio**	0.14	**0.152**
	**Tebuconazole**	0.012	
	Prochloraz	0.20	
	Chloorthalonil	0.024	
	Folpet	0.015	
	Carbendazim	0.056	
	Imidacloprid	0.024	
	Pyraclostrobine	0.027	
Sample 14	**Tebuconazole**	0.01	**0.010**
	Prochloraz	0.40	
	Captan	0.32	
	Chloorprofam	0.043	
	Folpet	0.068	
	Procymidon	0.021	

	Boscalid	0.013	
	Carbendazim	0.28	
	Daminozide	8.0	
	Pyraclostrobine	0.48	
	Spirotetramat	0.061	
Sample 15	Prochloraz	0.21	0
	Pyraclostrobine	0.027	
	Boscalid	0.019	
Sample 16	**Prothioconazole-desthio**	0.082	**0.094**
	**Tebuconazole**	0.012	
	Prochloraz	0.016	
	Bixafen	0.017	
	Fluopyram	0.04	
Sample 18	**Prothioconazole-desthio**	0.028	**0.048**
	**Tebuconazole**	0.02	
	Captan	0.76	
	Folpet	1.70	
	Procymidon	0.07	
	Prochloraz	0.59	
	Quintozeen	0.036	
	Boscalid	0.12	
	Carbendazim	0.21	
	Pyraclostrobine	1.80	
	Thiofanaat-methyl	1.10	
Sample 19	**Prothioconazole-desthio**	0.22	**0.246**
	**Tebuconazole**	0.015	
	**Thiabendazole**	0.011	
	Prochloraz	1.10	
	Captan	1.0	
	Chloorprofam	0.01	
	Chloorthalonil	0.097	
	Folpet	0.45	
	Metolachloor-S	0.029	
	Pendimethalin	0.018	
	Pirimifos-methyl	0.033	
	Carbendazim	0.42	
	Fluazinam	1.40	
	Imidacloprid	0.024	
	Metamitron	0.048	
	Pyraclostrobine	0.35	
	Thiofanaat-methyl	10.0	
	**Prothioconazole-desthio**	0.11	**0.122**
Sample 20	**Tebuconazole**	0.012	

	Prochloraz	0.80	
	Captan	0.38	
	Chloorthalonil	0.041	
	Folpet	0.051	
	Metolachloor-S	0.017	
	Pendimethalin	0.01	
	Carbendazim	0.17	
	Fluazinam	0.52	
	Imidacloprid	0.03	
	Pyraclostrobine	0.25	
	Spirotetramat	0.02	
	Thiofanaat-methyl	1.80	
Sample 21	**Prothioconazole-desthio**	**0.073**	**0.089**
	**Tebuconazole**	**0.016**	
	Bixafen	0.011	
	Fluopyram	0.033	
	Prochloraz	0.029	
	Thiofanaat-methyl	0.017	
Sample 22	Captan	0.26	0
	Folpet	0.024	
	Carbendazim	0.089	
	Pyraclostrobine	0.03	
	Spirotetramat	0.015	
Sample 23	**Prothioconazole-desthio**	0.11	**0.440**
	**Tebuconazole**	0.33	
	Prochloraz	15.0	
	Captan	0.45	
	Folpet	0.22	
	Boscalid	0.086	
	Carbendazim	1.10	
	Fluazinam	0.21	
	Pyraclostrobine	0.082	
	Thiofanaat-methyl	26.0	
Sample 37	**Epoxiconazole**	0.011	**0.076**
	**Prothioconazole-desthio**	0.05	
	**Tebuconazole**	0.015	
	Prochloraz	4.80	
	Captan	93.0	
	Folpet	150.0	
	Pendimethalin	0.036	
	Pirimifos-methyl	0.20	
	Procymidon	0.16	
	Quintozeen	0.026	

	Boscalid	0.03	
	Carbendazim	0.82	
	Fluazinam	1.80	
	Fluxapyroxad	0.019	
	Metamitron	0.012	
	Pyraclostrobine	5.70	
	Thiofanaat-methyl	8.0	
Sample 38	**Prothioconazole-desthio**	0.027	**0.041**
	**Tebuconazole**	0.014	
	Prochloraz	1.0	
	Captan	0.19	
	Folpet	0.035	
	Boscalid	0.024	
	Carbendazim	0.60	
	Fluazinam	0.086	
	Imidacloprid	0.76	
	Pyraclostrobine	1.60	
	Spirotetramat	0.024	
	Thiofanaat-methyl	0.78	
Sample 39			
	**Prothioconazole-desthio**	0.16	**0.172**
	**Tebuconazole**	0.012	
	Prochloraz	0.12	
	Captan	0.17	
	Folpet	0.016	
	Carbendazim	0.10	
	Thiofanaat-methyl	0.041	
Sample 40	**Tebuconazole**	0.018	**0.018**
	Prochloraz	0.14	
	Pyraclostrobine	0.042	
	Thiofanaat-methyl	0.015	
Sample 41	**Prothioconazole-desthio**	**0.073**	**0.073**
	Prochloraz	0.094	
	Carbendazim	0.011	
	Thiofanaat-methyl	0.018	
Sample 42	**Tebuconazole**	0.012	**0.012**
	Prochloraz	0.053	
	Boscalid	0.025	
	Carbendazim	0.024	
Sample 45	**Prothioconazole-desthio**	0.13	**0.272**
	**Tebuconazole**	0.012	
	**Thiabendazole**	0.13	
	Prochloraz	1.10	

	Captan	0.75	
	Chloorthalonil	0.01	
	Folpet	0.44	
	Azoxystrobin	0.02	
	Carbendazim	0.33	
	Fluazinam	0.021	
	Fluopyram	0.021	
	Imidacloprid	0.14	
	Pyraclostrobine	0.82	
Sample 46	**Tebuconazole**	0.018	**0.018**
	Prochloraz	0.07	
	Pyraclostrobine	0.098	
Sample 48	**Tebuconazole**	0.017	**0.017**
	Prochloraz	0.42	
	Procymidon	0.012	
	Quintozeen	0.056	
	Fluazinam	0.063	
	Pyraclostrobine	0.34	
Sample 49	**Prothioconazole-desthio**	0.05	**0.050**
	Prochloraz	0.053	
	Captan	1.40	
	Carbendazim	0.16	
	Spirotetramat	0.08	
	Thiofanaat-methyl	0.97	
Sample 51	**Prothioconazole-desthio**	0.01	**0.010**
	Prochloraz	1.50	
	Captan	3.10	
	Carbendazim	0.77	
	Fluazinam	0.057	
	Fluopyram	0.01	
	Thiofanaat-methyl	4.30	
Sample 52	**Prothioconazole-desthio**	0.22	**0.233**
	**Tebuconazole**	0.013	
	Prochloraz	0.14	
	Metolachloor-S	0.016	
	Carbendazim	0.11	
	Fluazinam	0.39	
	Pyraclostrobine	0.13	
	Spirotetramat	0.026	
	Thiofanaat-methyl	0.54	
Sample 54	**Prothioconazole-desthio**	0.01	**0.010**
	Imidacloprid	0.034	
	Prochloraz	0.11	

	Pyraclostrobine	0.02	
Sample 55	**Prothioconazole-desthio**	0.017	**0.028**
	**Tebuconazole**	0.011	
	Prochloraz	0.05	
	Fluopyram	0.13	
Sample 56	**Prothioconazole-desthio**	0.032	**0.048**
	**Tebuconazole**	0.016	
	Prochloraz	0.034	
	Boscalid	0.015	
	Fluopyram	0.10	
	Thiofanaat-methyl	0.013	
Sample 57	**Prothioconazole-desthio**	0.017	**0.177**
	**Tebuconazole**	0.16	
	Prochloraz	0.22	
	Captan	0.10	
	Metalaxyl	0.034	
	Azoxystrobin	0.37	
	Boscalid	0.087	
	Carbendazim	0.018	
	Fluazinam	0.014	
	Fluopyram	0.42	
	Imidacloprid	0.066	
	Pyraclostrobine	1.80	
	Teflubenzuron	0.083	
	Thiofanaat-methyl	0.043	
Sample 62			
	Chloorthalonil-4-hydroxy	0.017	**1.721**
	Prochloraz	0.082	
	**Prothioconazole**	1.700	
	**Prothioconazole-destio**	0.021	
Sample 77			
	Prochloraz	0.035	**2.836**
	**Prothioconazole**	2.800	
	**Prothioconazole-destio**	0.036	
Sample 94			
	Prochloraz	0.030	**2.330**
	**Prothioconazole**	2.300	
	**Prothioconazole-destio**	0.030	

Supplementary Table 3

Genetic analysis of the *cyp*51A gene and its promotor region, indicating the mechanism of azole resistance of isolates from various samples.

**Sample number**	**Resistance selection condition**	**Isolates**	**Date**	**Sampling sites**	*cyp*51A gene genotype
5	TEB	5-1	7-Jul-16	A	TR_{46}/Y121F/T289A
	TEB	5-2			TR_{46}/Y121F/T289A
	ITR	5-3			TR_{46}/Y121F/T289A
8	TEB	8-2	7-Jul-16	C	TR_{46}/Y121F/M172I/T289A/G448S
	ITR	8-3			TR_{46}/Y121F/T289A
	ITR	8-4			TR_{46}/Y121F/T289A
11	TEB	11-1	2-Aug-16	A	TR_{34}/L98H
	TEB	11-2			TR_{46}/Y121F/T289A
	ITR	11-3			TR_{34}/L98H
	ITR	11-4			TR_{46}/Y121F/T289A
13	TEB	13-1	20-Aug-16	B	TR_{46}/Y121F/T289A
	TEB	13-2			TR_{46}^{3}/Y121F/M172I/T289A/G448S
	ITR	13-3			TR_{46}/Y121F/T289A
	ITR	13-4			TR_{46}^{3}/Y121F/M172I/T289A/G448S
18	TEB	18-1	14-Sep-16	A	TR_{46}/Y121F/M172I/T289A/G448S
	ITR	18-3			TR_{46}/Y121F/M172I/T289A/G448S
	ITR	18-4			TR_{46}/Y121F/T289A
19	TEB	19-2	14-Sep-16	B	TR_{46}/Y121F/T289A
	ITR	19-3			TR_{34}/L98H
	ITR	19-4			TR_{46}/Y121F/T289A
25	TEB	25-1	14-Sep-16	C	TR_{46}/Y121F/T289A
	ITR	25-3			TR_{46}/Y121F/T289A
26	TEB	26-1	6-Oct-16	B	TR_{46}/Y121F/T289A
	TEB	26-2			TR_{46}/Y121F/T289A
	ITR	26-3			TR_{46}/Y121F/T289A
	ITR	26-4			TR_{34}/L98H
37	TEB	37-1	25-Oct-16	A	TR_{34}/L98H
	TEB	37-2			TR_{46}/Y121F/T289A
	ITR	37-3			TR_{46}/Y121F/T289A
	ITR	37-4			TR_{46}/Y121F/T289A
38	TEB	38-1	25-Oct-16	C	TR_{46}/Y121F/T289A
	ITR	38-3			TR_{46}/Y121F/T289A
	ITR	38-4			TR_{46}/Y121F/T289A
39	TEB	39-1	16-Oct-16	B	TR_{46}/Y121F/T289A
	TEB	39-2			TR_{46}^{4}/Y121F/M172I/T289A/G448S
	ITR	39-3			TR_{34}/L98H/S297T
	ITR	39-4			TR_{46}/Y121F/T289A

40	TEB	40-1	18-Nov-16	A	TR_{46}/Y121F/T289A/S363P/I364V/G448S
	TEB	40-2			TR_{46}/Y121F/T289A
47	TEB	47-1	30-Jan-17	A	TR_{46}/Y121F/T289A
	TEB	47-2			TR_{46}/Y121F/T289A
	ITR	47-4			TR_{34}/L98H/S297T
	ITR	47-3			TR_{46}/Y121F/T289A
48	TEB	48-1	24-Feb-17	A	TR_{46}/Y121F/T289A
	TEB	48-2			TR_{46}/Y121F/T289A
	ITR	48-3			TR_{46}/Y121F/T289A
	ITR	48-4			TR_{46}/Y121F/T289A
52	TEB	52-1	5-Jan-17	B	TR_{46}/Y121F/T289A
	TEB	52-2			TR_{46}/Y121F/T289A/S363P/I364V/G448S
	ITR	52-4			TR_{46}/Y121F/T289A
55	TEB	55-1	8-Mar-17	C	TR_{46}/Y121F/T289A
	TEB	55-2			TR_{46}/Y121F/T289A
	ITR	55-3			TR_{34}/L98H
	ITR	55-4			TR_{34}/L98H
62	TEB	62-1	9-Dec-16	C	TR_{46}/Y121F/T289A
	TEB	62-2			TR_{46}/Y121F/T289A
	TEB	62-3			TR_{46}/Y121F/T289A
	ITR	62-4			TR_{46}^{3}/Y121F/M172I/T289A/G448S
	ITR	62-5			TR_{46}/Y121F/T289A
	ITR	62-6			TR_{46}/Y121F/T289A
66	TEB	66-1	9-Dec-16	A	TR_{46}/Y121F/T289A
	TEB	66-2			TR_{46}/Y121F/T289A
	ITR	66-3			TR_{46}/Y121F/T289A
	ITR	66-4			TR_{34}/L98H
70	TEB	70-1	27-Mar-17	B	TR_{46}/Y121F/T289A/S363P/I364V/G448S
	TEB	70-2			TR_{46}/Y121F/T289A
	ITR	70-3			TR_{46}/Y121F/T289A
74	TEB	74-1	29-Apr-17	C	TR_{46}/Y121F/T289A
	ITR	74-3			TR_{46}/Y121F/T289A
	TEB	74-4			TR_{46}/Y121F/T289A
77	TEB	77-1	10-May-17	B	TR_{46}/Y121F/T289A
	TEB	77-2			TR_{46}/Y121F/T289A
	ITR	77-3			TR_{46}/Y121F/T289A/S363P/I364V/G448S
	ITR	77-4			TR_{46}/Y121F/T289A
78	TEB	78-1	10-May-17	C	TR_{46}/Y121F/T289A
	TEB	78-2			TR_{34}/L98H
	ITR	78-3			TR_{34}/L98H
80	ITR	80-1	22-May-17	C	TR_{46}/Y121F/T289A
81	TEB	81-1	22-May-17	C	TR_{46}/Y121F/T289A
	TEB	81-2			TR_{46}/Y121F/T289A
	ITR	81-3			TR_{46}/Y121F/T289A
	ITR	81-4			TR_{46}/Y121F/T289A

84	TEB	84-1	22-May-17	C	TR_{46}/Y121F/T289A
	TEB	84-2			TR_{34}/L98H
	ITR	84-3			TR_{34}/L98H
89	ITR	89-2	1-May-17	A	TR_{46}/Y121F/T289A
91	TEB	91-1	1-Jun-17	B	TR_{46}/Y121F/T289A
	ITR	91-4			TR_{46}/Y121F/T289A
92	TEB	92-1	1-Jun-17	C	TR_{46}^{3}/Y121F/M172I/T289A/G448S
	ITR	92-2			TR_{46}/Y121F/T289A
93	TEB	93-1	16-Jun-17	C	TR_{46}/Y121F/T289A
	ITR	93-2			TR_{46}/Y121F/T289A
94	TEB	94-1	16-Jun-17	B	TR_{46}/Y121F/T289A/S363P/I364V/G448S
	TEB	94-2			TR_{46}/Y121F/T289A
	ITR	94-4			TR_{46}/Y121F/T289A
95	TEB	95-1	3-Jul-17	A	TR_{46}/Y121F/T289A
	ITR	95-2			TR_{34}/L98H
96	TEB	96-1	3-Jul-17	C	TR_{34}/L98H
	ITR	96-2			TR_{46}/Y121F/T289A
97	TEB	97-1	3-Jul-17	B	TR_{46}/Y121F/T289A/S363P/I364V/G448S
	TEB	97-2			TR_{46}/Y121F/T289A
	ITR	97-3			TR_{46}/Y121F/T289A
	ITR	97-4			TR_{46}/Y121F/T289A/S363P/I364V/G448S
98	TEB	98-1	11-Jul-17	C	TR_{46}/Y121F/T289A
	TEB	98-2			TR_{46}/Y121F/T289A
	ITR	98-4			TR_{46}/Y121F/T289A
99	TEB	99-1	11-Jul-17	B	TR_{46}/Y121F/T289A/S363P/I364V/G448S
	ITR	99-2			TR_{46}/Y121F/T289A
100	TEB	100-1	11-Jul-17	A	TR_{34}/L98H
	ITR	100-2			TR_{34}/L98H
101	TEB	101-1	21-Jul-17	B	TR_{46}/Y121F/T289A/S363P/I364V/G448S
102	TEB	102-1	21-Jul-17	C	TR_{46}/Y121F/T289A
	TEB	102-2			TR_{46}/Y121F/M172I/T289A/G448S
	ITR	102-3			TR_{46}/Y121F/T289A
	ITR	102-4			TR_{34}/L98H
103	TEB	103-1	21-Jul-17	A	TR_{46}/Y121F/T289A
	ITR	103-2			TR_{46}/Y121F/T289A

Supplementary Table 4 statistics analysis

Since the data are not normally distributed, we performed a series non-parametric Spearman correlation tests, testing for correlations among the different environmental parameters. Please see below

Correlations

			azole	Total CFU	TEBf	ITRf	Collecting date	Farm
Spearman's rho	Azole level	Correlation Coefficient	1.000	**-.261**	**.101**	**-.010**	**-.035**	**-.060**
		Sig. (2-tailed)	.	.119	.552	.952	.837	.723
		N	37	37	37	37	37	37
	Total CFU	Correlation Coefficient	**-.261**	**1.000**	**.278**	**.306**	**.271**	**-.033**
		Sig. (2-tailed)	.119	.	.096	.066	.105	.846
		N	37	37	37	37	37	37
	TEBf	Correlation Coefficient	**.101**	**.278**	**1.000**	**.904****	**.046**	**-.300**
		Sig. (2-tailed)	.552	.096	.	.000	.787	.071
		N	37	37	37	37	37	37
	ITRf	Correlation Coefficient	**-.010**	**.306**	**.904****	**1.000**	**-.042**	**-.372***
		Sig. (2-tailed)	.952	.066	.000	.	.804	.023
		N	37	37	37	37	37	37
	Collecting date	Correlation Coefficient	**-.035**	**.271**	**.046**	**-.042**	**1.000**	**.209**
		Sig. (2-tailed)	.837	.105	.787	.804	.	.216
		N	37	37	37	37	37	37
	Farm	Correlation Coefficient	**-.060**	**-.033**	**-.300**	**-.372***	**.209**	**1.000**

	Sig. (2-tailed)	.723	.846	.071	.023	.216	.
	N	37	37	37	37	37	37

**. Correlation is significant at the 0.01 level (2-tailed).

*. Correlation is significant at the 0.05 level (2-tailed).

We found mostly weak and non-significant pair-wise correlations between azole level in the samples, total CFU, resistance frequency (to TEB, ITR), collection date and the farm at which the samples were collected. The only statistical correlation is between resistance frequency to TEB (TEBf) and and resistance frequency to ITR (ITRf); Correlation Coefficient = 0.904, P=0.023.

Supplementary Table 5

From all 117 isolates, we have selected a total of 32 isolates representative of all resistance mechanisms we detected, choosing 3 isolates (if the number was lower than 3, we choose all) per farm per genotype (resistance mechanism) for the standardized resistance EUCAST assay, see table below.

**Tandem repeat (promoter region)**	**Point mutation(s) (coding gene)**	A	B	C	Total
TR_{34}	L98H	3	2	3	8
TR_{34}	L98H/S297T	1	1	0	2
TR_{46}	Y121F/T289A	3	3	3	9
TR_{46}	Y121F/M172I/T289A/G448S	2	0	2	4
TR_{46}	Y121F/T289A/S363P/I364V/G448S	1	3	0	4
TR_{46}^{3}	Y121F/M172I/T289A/G448S	0	2	2	4
TR_{46}^{4}	Y121F/M172I/T289A/G448S	0	1	0	1
					32
